# PEGylated Domain I of Beta-2-Glycoprotein I Inhibits the Binding, Coagulopathic, and Thrombogenic Properties of IgG From Patients With the Antiphospholipid Syndrome

**DOI:** 10.3389/fimmu.2018.02413

**Published:** 2018-10-22

**Authors:** Thomas C. R. McDonnell, Rohan Willis, Charis Pericleous, Vera M. Ripoll, Ian P. Giles, David. A. Isenberg, Allan R. Brasier, Emilio B. Gonzalez, Elizabeth Papalardo, Zurina Romay-Penabad, Mohammad Jamaluddin, Yiannis Ioannou, Anisur Rahman

**Affiliations:** ^1^Division of Medicine, Centre for Rheumatology Research, University College London, London, United Kingdom; ^2^Internal Medicine, University of Texas Medical Branch, Galveston, TX, United States; ^3^Arthritis Research UK Centre for Adolescent Rheumatology, UCL/UCLH/Great Ormond Street Hospital, London, United Kingdom

**Keywords:** antiphospholipid syndrome, PEGylation, domain I, therapeutics, biologics

## Abstract

APS is an autoimmune disease in which antiphospholipid antibodies (aPL) cause vascular thrombosis and pregnancy morbidity. In patients with APS, aPL exert pathogenic actions by binding serum beta-2-glycoprotein I (β2GPI) via its N-terminal domain I (DI). We previously showed that bacterially-expressed recombinant DI inhibits biological actions of IgG derived from serum of patients with APS (APS-IgG). DI is too small (7 kDa) to be a viable therapeutic agent. Addition of polyethylene glycol (PEGylation) to small molecules enhances the serum half-life, reduces proteolytic targeting and can decrease immunogenicity. It is a common method of tailoring pharmacokinetic parameters and has been used in the production of many therapies in the clinic. However, PEGylation of molecules may reduce their biological activity, and the size of the PEG group can alter the balance between activity and half-life extension. Here we achieve production of site-specific PEGylation of recombinant DI (PEG-DI) and describe the activities *in vitro* and *in vivo* of three variants with different size PEG groups. All variants were able to inhibit APS-IgG from: binding to whole β2GPI in ELISA, altering the clotting properties of human plasma and promoting thrombosis and tissue factor expression in mice. These findings provide an important step on the path to developing DI into a first-in-class therapeutic in APS.

## Introduction

Antiphospholipid syndrome (APS) is an autoimmune disease in which antiphospholipid antibodies (aPL) interact with phospholipid/protein complexes in the body to cause vascular thrombosis and/or pregnancy morbidity (1). The exact population prevalence of APS is unknown but it has been estimated that aPL may contribute to 6.1% of cases of pregnancy morbidity, 13.5% of strokes, 11.5% of myocardial infarctions, and 9.5% of deep vein thromboses (DVT) (2). The only evidence-based treatment to prevent recurrent thrombosis in patients with APS is long-term anti-coagulation, generally with warfarin (3) though more recently new oral anticoagulants such as rivaroxaban have been used (4). Utility of these new oral anticoagulants however, is not yet fully established in treatment of thrombotic APS. In APS pregnancies, the standard of care remains daily treatment with oral aspirin plus subcutaneous heparin (5). Unfortunately recent data from the TRAPS trial has been disappointing with thrombosis rates in patients on Rivaroxaban being significantly higher (19 vs. 3%) (6). There is currently no therapeutic agent that targets the biologic actions of aPL specifically.

The key autoantigen targeted by pathogenic aPL is beta-2-glycoprotein I (β2GPI) (7), which consists of five domains [Domain I to Domain V (DI to DV)]. The N-terminal DI carries the key epitope recognized by aPL (8, 9). It is believed that the aPL-β2GPI complex interacts with the surface of target cells by means of binding between DV and negatively charged phospholipids on the cell membrane (10). This interaction leads to activation of cell surface receptors and intracellular signaling cascades causing changes in cellular behavior that promote thrombosis and/or pregnancy morbidity (10). Activation of target endothelial cells (ECs), monocytes and platelets by aPL induces the upregulation of several proinflammatory cytokines, key among them being tissue factor (TF) (11, 12). In our mouse thrombosis model utilizing knockout mice, we have previously shown that aPL-mediated increases in inflammatory cytokines, particularly TF levels, correlate with increased thrombus formation *in vivo* (13).

Our hypothesis is that an agent containing DI alone could inhibit binding of the pathogenic aPL to whole β2GPI (14). We have developed a method of expressing recombinant human DI in bacteria and have optimized this method to obtain high yield and purity of DI (15). We and others previously showed that recombinant DI inhibits binding of polyclonal IgG from patients with APS (APS-IgG) to whole human β2GPI (8, 9). We have also shown that recombinant DI inhibits thrombosis induced by human APS-IgG in a murine model (16). In these experiments, we tested wild-type DI as well as two variants created by site-directed mutation. One variant, with mutations of aspartic acid to serine at position 8 and glycine at position 9 (D8S,D9G) retained the ability to block both binding and thrombogenic properties of APS-IgG (16). We therefore decided to pursue the development of both wild-type DI and DI (D8S,D9G) as potential therapeutic agents for APS.

The small size (7 kDa) of DI would lead to a short half-life *in-vivo*, making it unsuitable as a therapeutic agent. There are a number of different ways to modify small biological molecules to improve half-life. These include addition of an immunoglobulin Fc region, as with the TNF blocking drug etanercept, and the chemical addition of polyethylene glycol groups (PEGylation). PEGylation has been used to develop a number of drugs used in rheumatic diseases notably pegloticase for gout and certolizumab pegol for rheumatoid arthritis [reviewed in 17)]. The advantages of PEGylation include improved half-life and reduced immunogenicity but potential disadvantages include loss of biological efficacy due to the large PEG group(s) blocking interaction of the protein with its physiological ligand (18–21). Larger PEG groups enhance half-life but reduce activity (19, 20, 22, 23) thus it is important first to assess activity of different PEGylated variants of a protein before going on to select a specific PEG size as successful development of a PEGylated therapeutic relies on efficiently balancing these two opposing conditions.

Site-directed PEGylation on disulfide bonds enables control of both the number and location of the PEG groups added to a protein, so that the properties of the PEGylated product are predictable and reproducible (24). DI has two disulfide bonds making it suitable for this method. The ideal product is monoPEGylated DI because two separate PEG groups attached to the same DI molecule would probably interfere with the ability of aPL to bind to their epitope (25–27).

In this paper we describe the PEGylation of both recombinant wild-type DI (WT-PEG-DI) and the D8S,D9G variant [PEG-DI (D8S,D9G)], and the characterization of their chemical and biological properties. We include a comparison of variants carrying PEG molecules of different sizes to facilitate future selection of a lead product to take forward to pharmacokinetic testing.

## Methods

A full description of the methods, including extended details of pH, media, buffers, and mouse experiments is included in Supplementary Material.

### Expression of recombinant DI and DI(D8S,D9G) in bacteria

Production was carried out as described previously (15). *E. coli* BL21^*^ cells were transfected with the recombinant DI plasmid and expression of DI was achieved by adding 1 mM IPTG followed by incubation with shaking overnight at 20°C. The bacteria were dissolved in lysis buffer, sonicated, and centrifuged to collect inclusion bodies containing the protein of interest. Inclusion bodies were dissolved and ground using a pestle and mortar into a chaotropic buffer before sonication (50% intensity, 50% cycles, 8 min) to increase solubilization. The expression plasmids are designed such that a nickel-binding hexahistidine tag is present at the N-terminal end of expressed DI, separated from it by a site for the protease Factor Xa (FXa) (15). The expressed protein from the solubilized inclusion bodies was therefore purified on a nickel column, re-folded in 0.6 M arginine buffer with a cysteine redox buffer (pH 8.5) and dialysed against 20 mM Tris, 0.1 M NaCl, pH 8. Protein was again purified post-folding using a nickel column and dialysed against phosphate buffered saline (PBS).

### PEGylation

Protein was reduced at a concentration of 0.4 mg/ml in 2 M arginine, 20 mM sodium phosphate (NaPO4, 0.1 M NaCl), 40 mM EDTA at pH 8.0 with 0.1 M DTT for 1 h at 20°C. This process was followed by removal of the reductant and buffer exchange on a PD-10 column to an identical buffer with 25 mM arginine rather than 2 M. PEGylation reagent was added (1:0.8 molar ratio) and incubated for 4 h at 4°C. This solution was then buffer exchanged to 20 mM sodium acetate with 0.05% Tween at pH 6.0 for cation exchange purification on a 5 ml SP-HP column (GE Healthcare) with a linear gradient from 20% buffer containing 1 M NaCl to 100% of the same buffer at 2 ml/min for 1 h. Fractions containing protein of the expected size of PEG-DI were identified by peaks on a chromatogram at 280 nm and then pooled. The hexahistidine tag was cleaved using FXa as in McDonnell et al. (15). Quantification from this point onwards was by BCA for both PEGylated and non-PEGylated form, thus 20 μg of WT-DI contains the same amount of DI as 20 μg WT-PEG-DI.

### Quantification

All concentrations of constructs were quantified using the BCA method. This method measures only the protein component of the construct (i.e., DI) and is unaffected by PEG. The concentrations expressed are based on protein concentration of the PEGylated constructs. This method was chosen as the form of quantification as previous work showed UV quantification was biased by the presence of a ring group in the PEGylation reagent. Thus, when we refer to 20 μg PEG-DI, we mean 20 μg of DI within the PEG-DI construct. Therefore, the PEGylated and non-PEGylated constructs contain the same amount of antigenic sites.

### Chemical characterization

Proteins were characterized for purity by reverse phase high performance liquid chromatography (RP-HPLC) using a C8 column with a linear gradient between 2% Acetonitrile (AcN), 0.05% trifluoroacetic acid (TFA), and 100% AcN 0.065% TFA. Proteins were also characterized by SDS PAGE for size.

### Production of APS-IgG samples from serum of patients

The clinical and serological data for the patients are shown in Table 1. IgG was purified from serum of these patients by passing these samples down a protein G column (Pierce). Eluted IgG was neutralized with 500 μl of 1 M Tris solution. Samples were then dialysed against PBS and total IgG content was quantified using a BCA assay.

**Table 1:** Clinical and serological data for patients with APS whose samples were used in these experiments.

	**APS patients**
Number	18
Age	49.5 (±12)
Female	13
Male	5
Caucasian	17
Asian	1
SLE/APS	8
VT	11
VT PM	6
CAPS	1
LA	17
Mean aCL (GPLU)	64 (*SD* 36)
Mean aB2GPI (GBU)	54 (*SD* 44)
Mean aDI (GDIU)	45 (*SD* 41)
aDI positive	10
aβ2GPI positive	12
aCL positive	13

*Patient data for those used to test for activity for D8S,D9G and WT protein. Note that cut-off for positivity in the aCL assay is >17GPLU, in the β2GPI assay it is >8GBU, and for DI positivity it is >10 GDIU. All cut off values were derived from 200 healthy patients*.

### Competitive inhibition anti-β2GPI ELISA

This ELISA was carried out as described previously (15). In brief, serum was diluted 1:50 in PBS/1% bovine serum albumin (BSA) and tested for binding in an anti-β2GPI ELISA. For each serum sample, the dilution level giving 50% of maximum binding in this assay was selected for use in the inhibition assay. Samples at this dilution were incubated with varying concentrations of a DI construct for 2 h at room temperature then tested again in the anti-β2GPI ELISA. The results were plotted as “% Binding remaining” on the y-axis against concentration of inhibitor on the x-axis where “% Binding remaining” = (Binding in presence of inhibitor)/(Binding in absence of inhibitor) × 100.

### Modified direct russell viper venom test (dRVVT)

Although APS is characterized by increased clotting caused by aPL, one of the tests used in clinical practice to detect aPL in serum is called the lupus anticoagulant (LA) test. The rationale of this test is that when clotting is triggered *in-vitro* using a reagent containing dilute Russell Viper Venom, the effect of adding aPL from a patient with APS is to inhibit clotting, prolonging the clotting time. This effect is reversed by adding an excess of phospholipids. The result is therefore expressed as the ratio of dRVVT-stimulated clotting time of the patient's plasma in the absence of phospholipid (LA-sensitive reagent or LS) to the clotting time in the presence of phospholipid (LA-resistant reagent or LR). A ratio above 1.1 suggests the presence of a circulating inhibitor of coagulation. This ratio was devised based on healthy control data, previously published studies and is suggested in the manufacturer kit insert.

We modified the dRVVT to test whether our PEG-DI products inhibits prolongation of the dRVVT clotting time caused by APS-IgG. APS-IgG samples were added to commercially available healthy human plasma at a concentration of 500 μg/ml for 15 min at 37°C before testing in the modified dRVVT assay. Those APS-IgG that gave LS/LR ratios >1.1 were used for inhibition assays. APS-IgG was incubated with inhibitor (DI or PEG-DI) at a 1:1 molar ratio with 50 μl of plasma for 15 min at 37°C. This mixture was then added to 350 μl of plasma and re-incubated for 15 min before testing for an LA-like effect. The outcome measure was ratio of clotting times seen in the presence of LS and LR reagents (LS/LR ratio). Reduction in this LS/LR ratio in the presence of DI or PEG-DI signified an inhibitory effect on the prolongation of the modified dRVVT caused by addition of APS-IgG. In control experiments, we used octreotide (a kind gift from Dr. Kozakowska of PolyTherics) or albumin (Sigma Aldrich) instead of DI or PEG-DI to exclude a non-specific effect of adding extraneous proteins to this assay.

### Passive transfer mouse model

The method was as described in previous papers (16). Male CD-1 mice (*n* = 5 per group) (Charles River Laboratories, Wilmington, MA) between 6 and 8 weeks in age were injected intraperitoneally (IP) with 500 μg in 1 ml APS-IgG in (all injections from a single preparation of IgG) and then 30–60 min later with either DI, PEG-DI conjugate or PBS control. IgG was isolated from a Caucasian 37 year old female APS/SLE patient with a history of pregnancy morbidity and venous thrombosis, serum levels of aCL, aβ2GPI, and aDI were 114 GPLU, 361 GBIU, and GDIU, respectively. IgG aβ2GPI levels were measured in the purified IgG as 132 SGU by INOVA Quantalite ELISA assay, aDI levels previously measured were 60 GDIU (at 100μg/ml). Negative control mice were injected IP with 500 μg in 1 ml normal healthy serum (NHS)-IgG. All materials had endotoxin levels below 1.5 EU/ml. These injections were repeated after 48 h and the thrombogenicity of aPL was assessed at 72 h after the first injection. At this time, mice were anesthetized and one of the femoral veins was exposed for observation with an approximate 0.5 mm segment trans-illuminated using a microscope equipped with a closed-circuit video system. The isolated vein segment was pinched to introduce a standardized injury and thrombus formation and dissolution was visualized and recorded. The treatment groups were as follows: APS-IgG + PBS control, NHS-IgG Alone, APS-IgG + 40 μg non-PEGylated WT-DI, APS-IgG + 40 μg non-PEGylated DI (D8S,D9G), APS-IgG + 40 μg 20 kDa PEGylated DI, APS-IgG + 40 μg 20 kDa PEGylated DI (D8S,D9G), APS-IgG + 20 μg 20 kDa PEGylated DI, PEG alone, and APS-IgG + PEG.

Three outcome measures were assessed, as fully described in previous papers and in Supplemental Data (16). These were:

a) Induced thrombus size: A total of three thrombi were generated in each mouse and the largest cross-sectional area of each thrombus during the formation-dissolution cycle was measured five times and a mean value calculated (in μm^2^).
b) Activity of tissue factor (TF) in peritoneal macrophages by a chromogenic assay. Results were standardized with reference to the protein concentration of lysates and expressed in pM/mg/ml.
c) Tissue Factor expression in mouse carotid homogenates [measured as described in (b) above].

All animals were housed in the viral antibody-free barrier facility at the University of Texas Medical Branch (UTMB). Animal use and care were in accordance with the UTMB Institutional Animal Care and Use Committee (IACUC) guidelines.

### Statistical analysis

Results were expressed as means plus or minus standard deviation as appropriate. A one way analysis of variance by ANOVA followed by the Tukey-multiple comparison test was used to compare differences among mouse groups. These analyses were performed using the xlStat. Statistics were also carried out in prism using ANOVA and *T*-Tests. Statistical analysis primarily compared PEGylated DI variants with their non-modified equivalent.

## Results

### WT-PEG-DI was produced at over 95% purity and monopegylated variants containing 20, 30, and 40 kDa PEG were obtained

**Figure 1:**
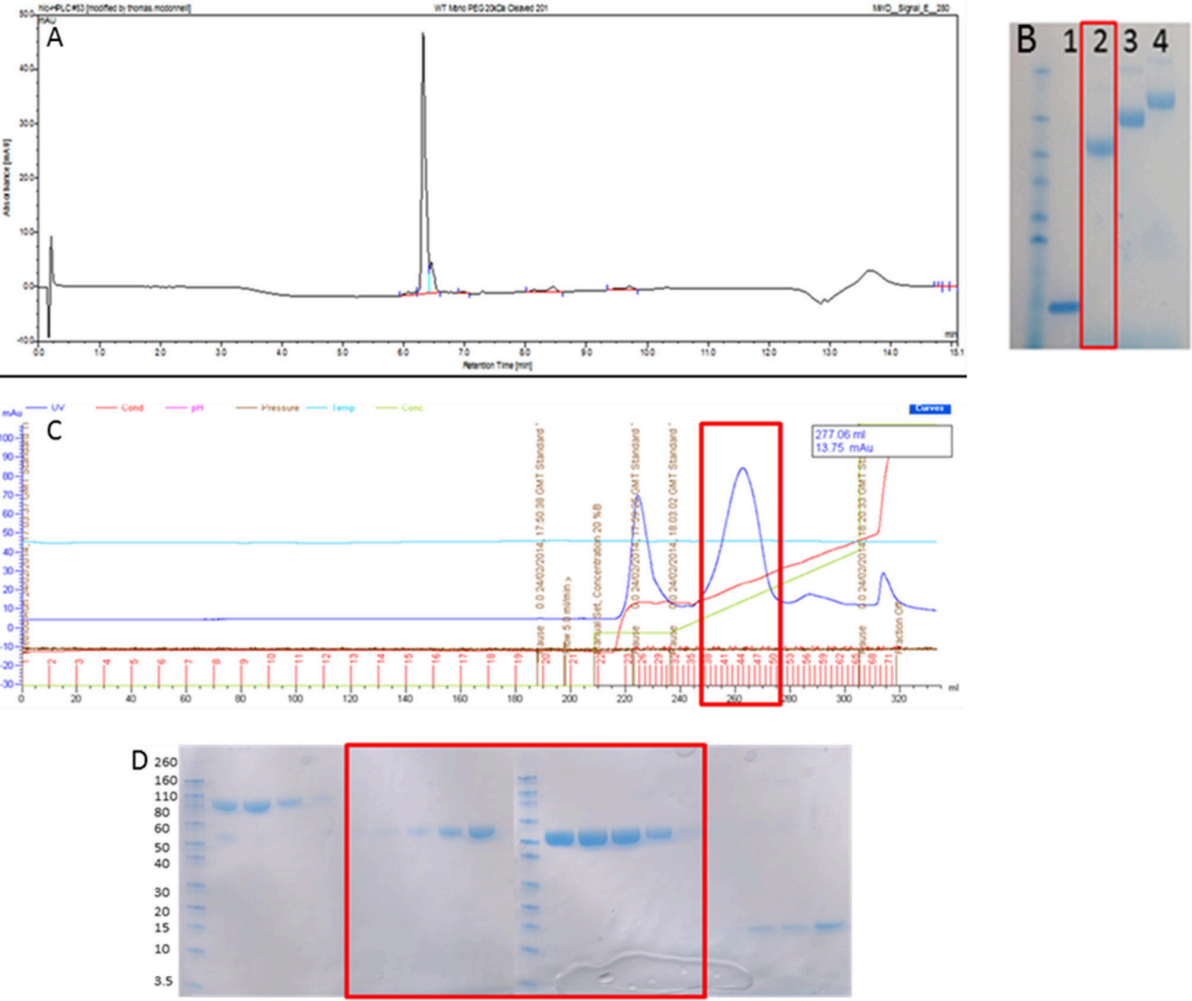
Production of monoPEGylated DI. **(A)** A chromatogram demonstrating the production of >95% pure conjugated WT-PEG-DI shown as a single peak by Reverse Phase HPLC using a C18 column. **(B)** SDS PAGE gel showing unmodified WT-DI (lane 1), WT-DI conjugated to 20 kDa PEG (lane 2), WT-DI conjugated to 30 kDa PEG (lane 3), and WT-DI conjugated to 40 kDa PEG (lane 4). The red box around lane 2 indicates the sample shown in chromatogram **(A)**. **(C)** Chromatogram showing the result of cation exchange purification to separate non-conjugated WT-DI from PEG-WT-DI. The peak at 225 ml is diPEGylated WT-DI. The large peak at 260 ml, highlighted by the red box, is monoPEGylated WT-DI. The small peak at 320 ml is residual non-PEGylated WT-DI. **(D)** SDS PAGE gel showing the different forms of WT-PEG-DI obtained from the cation exchange purification **(C)**. The Bands in the three lanes at the far left are diPEGylated WT-DI. Bands in the center, highlighted by the red box, are monoPEGylated WT-DI. The faint bands in the lanes on the far right are residual non-PEGylated WT-DI.

As can be seen in Figures 1A,B, 20 kDa WT-PEG-DI, 30 kDa WT-PEG-DI, and 40 kDa WT-PEG-DI were generated and the purity of this product exceeded 95%. Since DI contains two disulfide bonds, the potential products of the reaction were diPEGylated DI, monoPEGylated DI, and residual non-PEGylated DI. The chromatogram in Figure 1C shows that monoPEGylated DI was the dominant product and could be easily separated in different fractions from the other two products. The fractions shown in the chromatogram were run on an SDS-PAGE gel (Figure 1D), which shows that monoPEGylated, diPEGylated, and non-PEGylated DI were all obtained separately.

### WT-PEG-DI retains the ability to inhibit binding of serum from patients with APS to whole β2GPI

**Figure 2:**
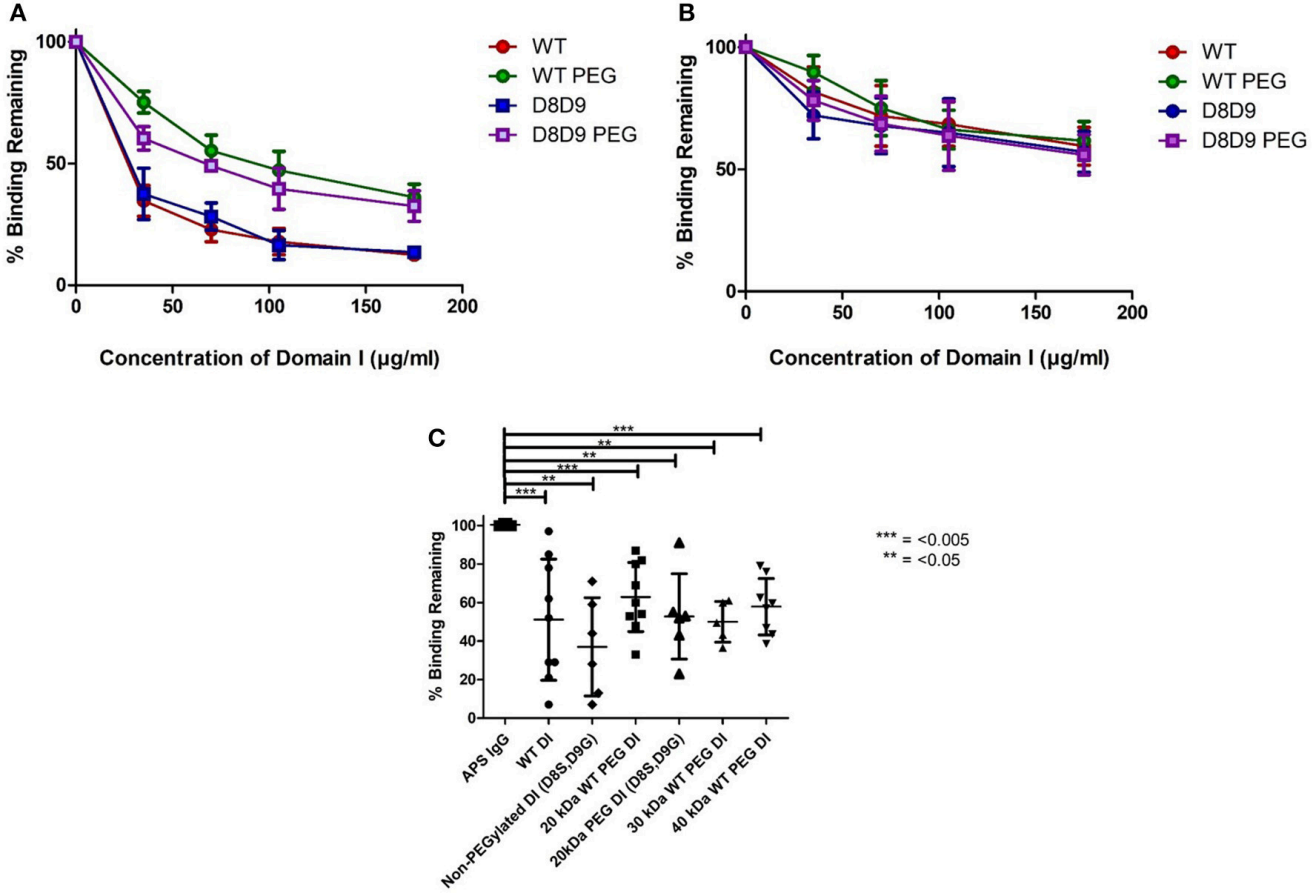
PEGylated DI blocks binding of IgG in APS serum samples to whole β2GPI. **(A)** This graph shows the combined results from inhibition ELISAs of serum samples from three patients whose binding to whole β2GPI was inhibited more strongly by non-PEGylated than by PEGylated DI constructs. **(A)** Contains patients with a high level of serum aDI antibodies (average >80 GDIU). **(B)** This graph shows the combined results from inhibition ELISAs of serum samples from three patients whose binding to whole β2GPI was inhibited equally by non-PEGylated and PEGylated DI constructs. These patients have a lower level of serum aDI (average < 45 GDIU). **(C)** The dot bot shows the combined results from testing samples from 9 patients [including all six from **(A,B)**]. The first column on the left shows binding to β2GPI in the absence of any inhibitor and the other columns show the binding in the presence of different inhibitors at a concentration of 100 μg/ml. For both WT-DI and DI(D8S,D9G), addition of 20 kDa PEG does not alter the inhibitory capacity of DI in this assay and the same is true for 30 and 40 kDa PEG in the case of WT-DI (these larger PEG sizes were not tested for DI(D8S,D9G). Significant differences were seen between the results obtained with APS IgG alone and those obtained with all inhibitors PEGylated or non-PEGylated (***p < 0.005, **p < 0.05) but no significant differences were seen between any of the inhibitors.

Figures 2A,B demonstrate the results obtained in the inhibition ELISA with serum samples from six patients with APS. Binding to β2GPI on the plate was inhibited by increasing concentrations of the following products; non-PEGylated wild-type DI (WT-DI), WT-PEG-DI, non-PEGylated DI (D8S,D9G), and PEG-DI (D8S,D9G). Greater inhibition is shown by lower curves, signifying that less of the original binding capacity was maintained in the presence of the inhibitor.

For most of the samples there is little difference between the DI and DI(D8S,D9) curves, whether PEGylated (compare Green with Purple), or non-PEGylated (compare Red with Blue) showing that introduction of the D8S,D9G mutations had little effect on inhibitory capacity of DI in this assay.

The effect of PEGylation, this can be seen by comparing the WT-DI (Red) to WT-PEG-DI (Green) and the DI(D8S,D9G) (Blue) to PEGylated DI(D8S,D9G) (Purple). The samples can be divided into two types. One type, exemplified by panel A was characterized by strong inhibition (80% or more) by the two non-PEGylated products whereas their PEGylated equivalents were still able to inhibit binding but not by as much (typically a maximum of 40–50%). The other type of patient, exemplified by panel B showed less inhibition of binding but this level of inhibition (typically a maximum of 30–50%) was similar for the non-PEGylated and PEGylated DI molecules. Of note, in direct anti-DI ELISA the type A samples had higher binding to DI (mean 87.5GDIU) than the type B samples (mean 44.4GDIU).

We then compiled all the patient data into a single graph displaying average inhibition within groups (Figure 2C). We utilized the 100 mcg dose as this concentration was before the plateau of inhibition; these data are shown in Figure 2C. It demonstrates that 20 kDa WT-PEG-DI (*n* = 10), 30 kDa WT-PEG-DI (*n* = 6), and 40-kDa WT-PEG-DI (*n* = 9) all inhibit binding to a similar level. When combining the results from testing multiple serum samples, there was no significant loss of inhibitory capacity with the PEGylated compared to the non-PEGylated variants.

### PEGylated DI retains the ability to inhibit the effect of APS-IgG in the modified dRVVT assay

The outcome measure in this assay is the ratio of clotting time in the presence of the lupus anticoagulant-sensitive (LS) reagent to clotting time in the presence of the lupus anticoagulant-resistant (LR) reagent—referred to in the figure as LS:LR ratio or ratio of reagents. As described in the methods section, a reduction in this ratio in the presence of DI or PEG-DI indicates inhibition of the prolongation of the dRVVT.

**Figure 3:**
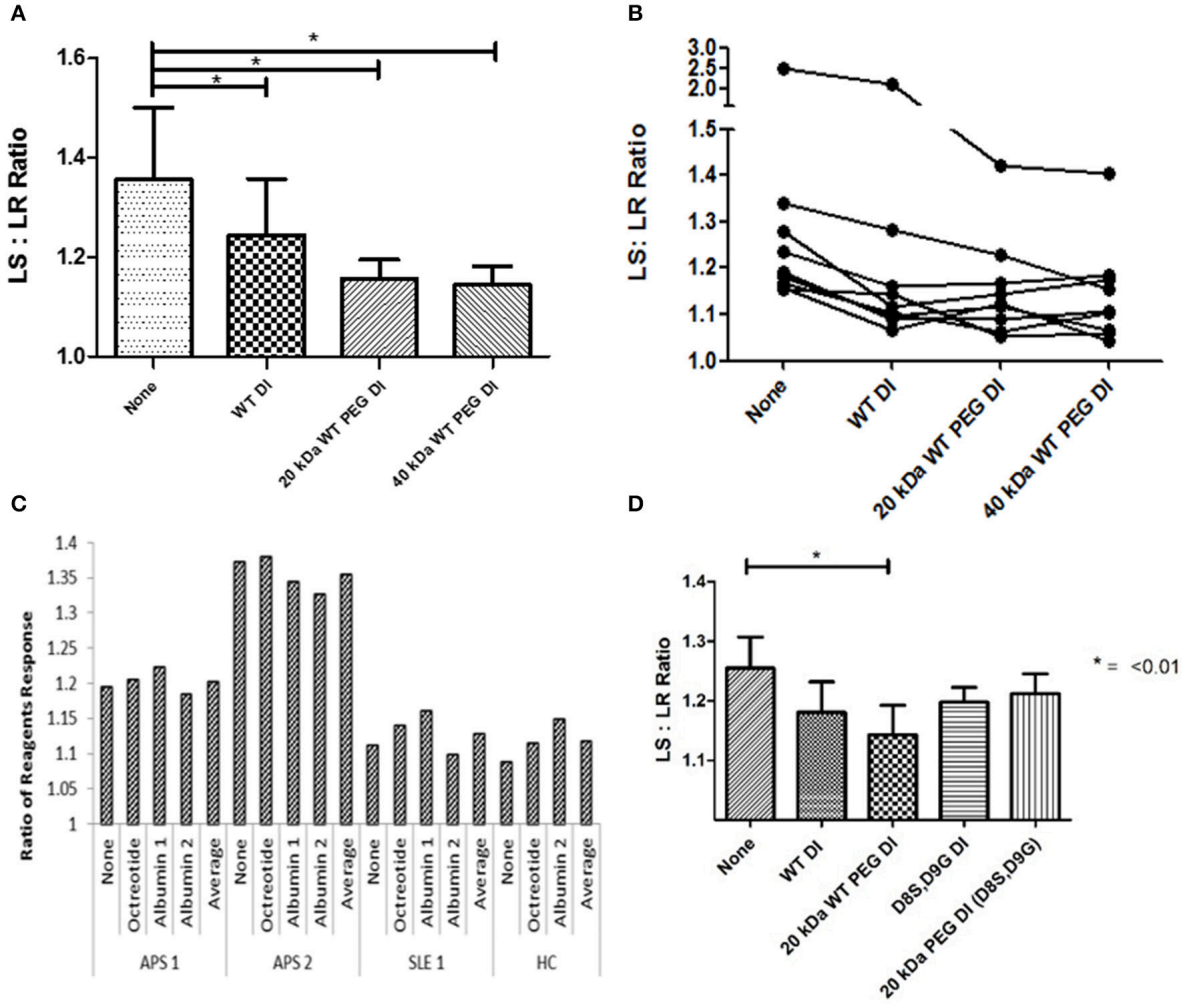
PEGylated DI inhibits the LA effect of APS-IgG samples. In each figure, the y-axis shows the ratio of clotting times obtained in the presence of LS and LR reagents. LS:LR value >1.1 suggests an LA effect. **(A)** Demonstration of the effect of inhibitors on a modified dRVVT assay, this graph shows combined results from testing 9 samples. The mean LS:LR ratio was significantly lower in the presence of non-PEG-DI, 20 or 40 kDa WT-PEG-DI than in the absence of any of these agents. Significance was seen with all inhibitors compared to no inhibition (*p < 0.05) but there were no significant differences between the results obtained with non-PEG-DI, 20 and 40-kDa-PEG-DI. **(B)** Each separate line shows results from IgG of an individual patient inhibited with PEGylated and non-PEGylated Domain I in the modified dRVVT assay. The LS:LR ratios obtained with PEG-DI are either similar to or lower than those obtained with non-PEG-DI for all samples. **(C)** Control experiment showing that addition of non-DI proteins (albumin and octreotide) has no effect on LS:LR ratios in this coagulation assay. Each group of five columns shows the results from blood of a single individual (two with APS, one with SLE but not APS, and one HC) and in each group there are no significant differences between the columns. **(D)** Comparison of WT-DI and DI(D8S,D9G) in tests on three samples. Here PEG-DI(D8S,D9G) and non-PEG-DI(D8S,D9G) have similar effects to each other but less than WT-PEG-DI.

Results from 9 samples can be seen in Figure 3A. There was significant inhibition (*P* < 0.05) with non-PEGylated WT-DI and both 20 kDa WT-PEG-DI and 40 kDa WT-PEG-DI. PEGylation did not reduce the ability of WT-DI to inhibit the action of APS-IgG in this assay. Furthermore, Figure 3B shows the results for each sample separately. For two samples, the PEGylated variants reduced LS:LR ratio more than non-PEG-DI. WT-PEG-DI showed no effect on the clotting of normal plasma in the absence of APS-IgG. PEG alone showed no inhibitory effects (data not shown).

Figure 3C shows the results of control experiments designed to show that only proteins containing DI inhibited APS-IgG in this assay. We added octreotide or two different preparations of albumin to either APS-IgG or control IgG preparations [from a patient with systemic lupus erythematosus (SLE) or a healthy subject]. The figure shows that neither albumin nor octreotide had any effect on the LS:LR ratio obtained with any of these samples.

Figure 3D shows the results of samples from three patients for which inhibitory effects of both WT-DI and DI(D8S,D9G) were tested. Both PEGylated and non-PEGylated forms of both these agents inhibited the LA-like effect of APS-IgG in these experiments.

### PEGylated DI retains the ability to inhibit the effect of APS-IgG on development of thrombosis in a mouse model of APS

**Figure 4:**
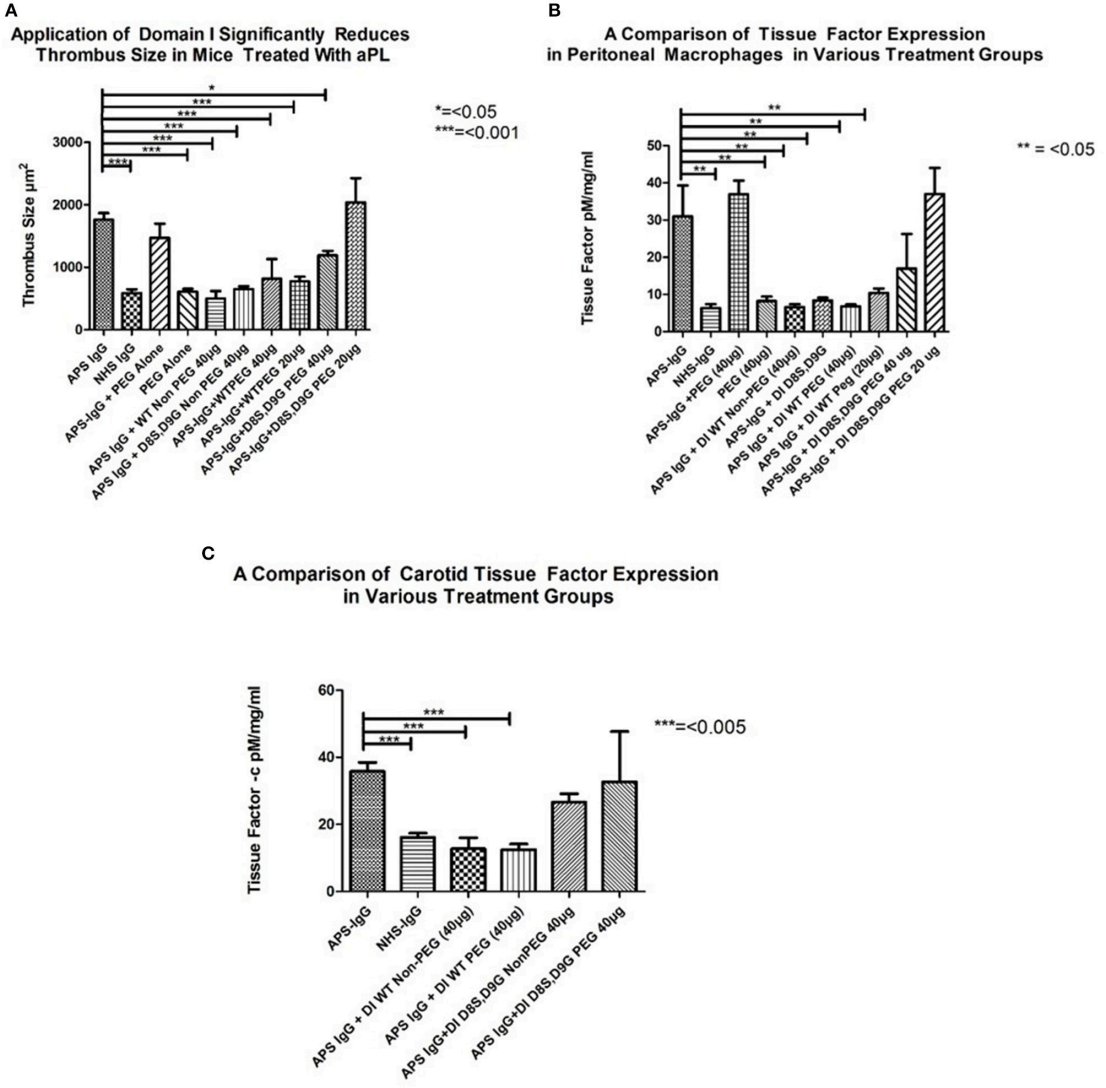
Inhibitory effects of non-PEG-DI and PEG-DI in a passive transfer mouse model of APS. Mice received either NHS-IgG (500 μg), APS-IgG (500 μg), or 40 μg PEG alone with no IgG. APS-IgG, but not NHS-IgG or PEG alone, stimulated increased thrombosis **(A)**, increased peritoneal macrophage TF expression **(B)**, and increased carotid TF expression **(C)**. The effects of the various PEGylated and non-PEGylated DI constructs are shown in the columns on the right of the graphs and explained fully in the results section.

In each panel of Figure 4, the two columns on the far left show the difference in outcome obtained after administering APS-IgG and NHS-IgG in the absence of any inhibitor. In Figure 4A the following two columns show that PEG alone does not cause enhanced thrombosis (compared to NHS-IgG) nor does it inhibit the thrombotic effect of APS-IgG. The next two columns compare the WT-DI and DI(D8S,D9G) non-PEGylated proteins for their ability to inhibit thrombosis; both significantly reduce thrombus size. The following two columns show that 20 KDa WT-PEG-DI also inhibits thrombosis, but not as well as non-PEGylated WT-DI, with little difference between the 20 and 40 μg doses. However, the two columns on the far right show a marked loss of inhibitory activity of DI(D8S,D9G) after PEGylation, especially at the 20 μg dose.

Figure 4B shows TF expression in peritoneal macrophages of mice exposed to APS-IgG in the presence of various inhibitors. The columns on the left (3–5) show inhibition by both PEGylated and non-PEGylated WT-DI whereas the columns on the right (8–10) show that the inhibitory effect of DI(D8S,D9G) in this assay is dramatically reduced after PEGylation. In fact, Figures 4A,B show that a dose of 20 μg PEG-DI(D8S,D9G) has no inhibitory effect at all. PEG in the absence of APS-IgG does not have any effect on TF expression in peritoneal macrophages.

As can be seen in Figure 4C both non-PEGylated WT-DI and WT-PEG-DI inhibit the expression of TF in mouse carotid exposed to APS-IgG by similar amounts. Conversely DI (D8S,D9G) does not significantly reduce carotid TF expression, whether or not it is PEGylated.

Since the mice in this model are subjected to anesthesia and a surgical procedure, some premature deaths were expected. All but one of these deaths occurred while mice were still under anesthesia. Thus, in the control groups that were not exposed to DI, 1/5 mice died in each of the following groups; APS-IgG alone, NHS IgG alone, and PEG alone and no mice died in the group of 7 mice given APS IgG and PEG. The results were similar in the mice given APS-IgG plus non-PEGylated WT-DI (1/5 died) and APS-IgG plus non-PEGylated DI (D8S,D9G) (0/5 died). Regarding the PEGylated constructs, two different doses of PEG-DI were tested. At high dose (40 μg) 2/5 mice died in both the APS-IgG plus PEG-WT-DI and the APS-IgG plus PEG- DI(D8S/D9G) group. However, at low dose (20 μg) 0/5 mice died in the APS-IgG plus PEG-WT-DI and 2/5 died in the APS-IgG plus PEG-DI(D8S/D9G) group.

We carried out analyses to investigate possible causes of toxicity in these mice. Mice treated with high dose of PEG-WT-DI and either dose of PEG-DI(D8S/D9G) displayed some signs of inflammatory damage localized to the peritoneal cavity manifesting as erythema, exudation, and in the case of some of the animals treated with the PEG-DI(D8S,D9G) agent, petechial hemorrhage. However, none of the mice exhibited any of the typical signs of a systemic inflammatory response, namely coat ruffling, impaired consciousness, activity, eye movements, respiration, or response to stimuli. Importantly, the mice exposed to low dose PEG-WT-DI had neither localized nor systemic inflammation and none of them died prematurely.

We measured the levels of the acute phase reactants interleukin-6 (IL-6) and tumor necrosis factor-α (TNF-α) in mice treated with the 40 μg dose of PEG-DI(D8S,D9G) or PEG-WT-DI and found that they were similar to those seen in mice treated with a low dose of endotoxin (0.001 mg), which does not cause systemic inflammation. These levels were ~10 to 60-fold less than those seen in mice treated with a typical endotoxin dose to induce sepsis (0.5 mg).

In summary, these mortality data showed that administration of the non-PEG-DI and low dose PEG-20kDaWT-DI did not demonstrate any excess deaths compared to control groups, whereas the PEG-20kDa DI(D8S/D9G) group had a slight excess (2/5 deaths compared to 1/5 in control groups) at either dose. Thus, the PEGylated mutant version of DI showed unfavorable properties in this *in-vivo* model compared to PEGylated WT-DI both in terms of activity (see above) and toxicity.

## Discussion

We previously showed that recombinant DI expressed in bacteria blocked the binding and thrombogenic actions of APS-IgG (16). Since DI alone is not a viable therapeutic agent due to its small size, we have pursued development of a PEGylated form of DI. The use of PEGylation is common in the production of therapeutics due to its positive pharmacokinetic effects, however, this is weighed against a loss of activity for the protein. For this reason it is essential to characterize protein activity to determine which size of PEG would be optimal in terms of balancing increased half-life with maximal activity, prior to testing pharmacokinetics. Equally, before proceeding to trials of efficacy, it was important to optimize the method of production of pure monoPEGylated DI. The optimization of expression of DI in *E. coli* has been reported in a previous paper (15) and the optimization of PEGylation and purification is reported here.

Total inhibition of binding of APS-IgG to β2GPI was not seen even at high concentrations of PEG-DI because the serum anti-β2GPI response in patients with APS is polyclonal and comprises antibodies to different domains of β2GPI. The anti-DI component of this polyclonal response is believed to play the predominant role in pathogenesis of the APS. Both a Dutch group de Laat et al. (28) and our group have shown that anti-DI levels are more closely associated with risk of thrombosis than anti-β2GPI or anti-cardiolipin antibody levels (29). Andreoli et al. tested serum from a variety of groups for antibodies to DI and to DIV/V (30). Whereas anti-DI and anti-DIV/V were equally likely to be found in subjects with no autoimmune disease, anti-DI was found four times more commonly than anti-DIV/V in autoimmune patients (30). In a mouse model, we showed that when APS-IgG was separated into anti-DI-rich and anti-DI-poor fractions by affinity-purification on a column carrying recombinant DI, the ability to cause vascular thrombosis was concentrated in the anti-DI rich fraction (31). Thus, we believe that 50% inhibition of binding of APS-IgG to β2GPI that we have demonstrated with PEG-DI is of potential clinical benefit, because the subpopulation of anti-β2GPI being inhibited is also the group with the major thrombogenic effect.

Our description of a modified dRVVT assay to test the ability of potential therapeutic agents to block the ability of APS-IgG to prolong the modified dRVVT is itself novel and could be used to test other potential therapeutic agents in APS, however, further testing is required before the modified dRVVT could be considered a reliable marker. Testing the LA-like effect is relevant as several studies have shown that LA may be a stronger predictor of clinical outcomes than the anti-cardiolipin or anti-β2GPI tests in patients with SLE and APS (32), particularly in pregnancy (33). WT-PEG-DI was at least as effective an inhibitor in this assay as non-PEG WT-DI. However, unlike the binding assay [where we obtained similar results with WT-DI and DI (D8S,D9G)] in the modified dRVVT assay PEGylation seems to reduce the efficacy of DI (D8S,D9G). Replacing two larger negatively charged amino acids with smaller neutral ones could alter exposure of the disulfide bond to which the PEG binds and might therefore alter the orientation of the PEG group itself, thus influencing factors such as steric hindrance.

In the mouse model experiments, it was also clear that WT–PEG-DI had advantages over PEG-DI (D8S,D9G). Whereas, both 20 μg WT-PEG-DI and 40 μg WT-PEG-DI inhibited the biological effects of APS-IgG on thrombosis and TF expression, the PEGylated forms of DI(D8S,D9G) had reduced efficacy as inhibitors. Furthermore, significant toxicity was seen with the PEG-DI(D8S,D9G) agent but not the WT-PEG-DI agent. While the 20 and 40 μg doses of the PEG-DI(D8S,D9G) agent produced inflammatory damage, resulting in premature deaths of 40% of the mice in each group, the WT-PEG-DI agent did not have this effect when given at a dose of 20 μg.

It was not possible to use the mouse model to establish half-lives of the DI or DI-PEG conjugates for several reasons. In this acute model, mice are sacrificed after 72 h, as such little data would be generated regarding the half-lives of protein from such a small exposure. Equally, DI or PEG-DI is bound by circulating antibody, this would alter the values detected if we attempted to use this model to characterize half-lives. The aim of the mouse model was to characterize the activity of the conjugates before establishing a lead candidate for therapeutic development, it is only through initially testing the activity of the protein conjugates that the correct PEG size can be tailored to the protein.

Although the ultimate purpose in PEGylating DI was to improve its serum half-life, we have not yet carried out pharmacokinetic experiments to prove that this has been achieved. Clearly these experiments must be done before clinical application of PEG-DI can be considered, furthermore, we also plan to test ability of PEG-DI to inhibit thrombus formation in a chronic mouse model of APS, in which mice are immunized with human β2GPI and thus develop their own anti-β2GPI antibodies (34) (as opposed to the passive transfer of human APS-IgG reported in this paper).

We are unsure of the exact mechanism of toxicity attributed to the PEGylated DI(D8S,D9G) but it seems clear that it is localized to the site of inoculation and given the changes in size and charge to the DI molecule imparted by the PEG conjugate, impaired absorption of the agent across the peritoneum may be a contributing factor. Importantly, we ruled out the formation of immune complexes (CIC) in the peritoneal cavity as a possible cause by measuring levels in collected peritoneal exudates (data not shown). Future toxicity studies will focus on determining specific effects of PEGylated agents when introduced in the intraperitoneal vs. intravascular compartments.

We hypothesize that the difference in activity *in-vitro* and toxicity *in-vivo* between the two conjugates may be due to a difference in the binding site of the PEG. The PEGylation technology used requires access to surface exposed disulphide bonds. The dual mutation in the DI(D8S,D9G) protein allows access to the disulphide from two sides whilst the WT-DI protein restricts access to a single site. The mutations may therefore allow a different PEGylation which in turn may lead to masking the active site. This difference in site could also explain the increased toxicity seen with the PEG-DI(D8S,D9G) agent as the difference in PEGylation site may also effect diffusion constants and solubility. This theory is, however, speculative and would require significant modeling work to fully confirm.

Overall, the results of our programme of experiments clearly identify WT-PEG-DI as a strong potential lead for APS and a better candidate than PEG-DI(D8S,D9G), though the optimal size of PEG cannot be identified until pharmacokinetic experiments have been carried out.

Limitations of the work include the fact that we have tested samples from a relatively small number of patients with APS. Particularly for the *in-vivo* experiments there are practical limitations to the number of different samples that can be tested, since large numbers of mice are required for tests on each APS-IgG sample. For the modified dRVVT assay we can only use samples that have LS:LR >1.1 in the absence of inhibitor. We have mitigated the problem of limited sample numbers by using three complementary assays to test efficacy of PEG-DI as an inhibitor of APS-IgG. We should stress however, PEG-DI may not be effective in all patients; potentially it may show greatest effect in patients with high anti-DI levels. Measuring anti-DI levels could identify the sub-population of patients with APS likely to respond to PEG-DI. A study by Pericleous et al. showed ~40% of patients with APS had aDI positivity (29), a finding mirrored by a recent review (35).

Possible limitations of PEG-DI include potential toxicity or immunogenicity of the PEG molecule. PEG is harmless to animals at concentrations as high as 16 g per kg [1,000-fold higher than the normal therapeutic dose of protein-PEG conjugates in humans (26)] but anti-PEG antibodies are found in between 8 and 25% of the healthy population (36, 37). This phenomenon is possibly due to use of PEG in everyday products such as hand creams. It is not clear whether those anti-PEG antibodies would affect efficacy of PEGylated drugs. Although anti-PEG and anti-drug antibodies adversely affected efficacy of PEGylated uricase used in gout, the same was not true in large clinical trials of certolizumab pegol, a PEGylated anti-tumor necrosis factor Fab used in rheumatoid arthritis. Though antibodies to this drug were seen in 5–6% of patients in the RAPID 1 (38) and RAPID 2 (39) clinical trials, there was no evidence that presence of these antibodies affected clinical outcomes.

In conclusion, there is a pressing need for targeted therapies in APS given that none have yet been developed for this disease. We have developed PEG-DI, an agent designed to block binding of pathogenic aPL to β2GPI, and have shown that it is effective at blocking the effects of APS-IgG in three different assays, two *in-vitro* and one *in-vivo*. These data support pharmacokinetic and toxicology experiments which are now underway to develop PEG-DI as a first-in-class therapeutic for APS.

## Ethics statement

This study was carried out in accordance with the recommendations of London Hampstead Research Ethics Committee Ref No 12/LO/0373 with written informed consent from all subjects. All subjects gave written informed consent in accordance with the Declaration of Helsinki. The protocol was approved by the London Hampstead Research Ethics Committee Ref No 12/LO/0373. All animal research was conducted in a responsible, humane manner under the guidance of the University of Texas Medical Branch Institutional Animal Care and Use Committee (UTMB IACUC). UTMB IACUC is a Category I institution accredited by the Association for Assessment and Accreditation of Laboratory Animal Care International (AAALAC) and is in full compliance with the US Federal Animal Welfare Act (1966) and Texas animal care laws.

## Author contributions

TM carried out laboratory work, developed methodology, and co-wrote the paper. RW carried out mouse work in Texas. CP and YI developed methodology and gave intellectual guidance. VR and IG gave intellectual guidance. DI, AB, and EG gave direction and support. EP, ZR-P, and MJ carried out practical work in Texas regarding mouse models. AR gave intellectual input, co-wrote the paper and supervised the project.

### Conflict of interest statement

TM, CP, YI, IG, and AR are named inventors on a Patent filed in the US for PEG-DI. The remaining authors declare that the research was conducted in the absence of any commercial or financial relationships that could be construed as a potential conflict of interest.
