# Supplementary material for: PEGylated Domain I of Beta-2-Glycoprotein I Inhibits the Binding, Coagulopathic, and Thrombogenic Properties of IgG From Patients With the Antiphospholipid Syndrome
Source: Front Immunol. 2018 Oct 22;9:2413. doi: 10.3389/fimmu.2018.02413 (PMC6204385; doi:10.3389/fimmu.2018.02413)
Supplement: Supplementary file 1 [file Presentation_1.pdf]

Supplementary Material

Expression of recombinant DI and DI(D8S,D9G) in bacteria:

Production was carried out as described previously (*11*). In summary, *E.coli* BL21* cells transfected with the recombinant expression plasmid were incubated in shaking baffled flasks to an optical density of 7, measured at 600 nm. Induction of expression from the plasmid was achieved by adding 1 mM IPTG followed by incubation with shaking overnight at 20 °C. The bacteria were harvested by hollowfibre (G.E Healthcare) before being washed with phosphate buffered saline at pH 7.4(PBS) and snap frozen on dry ice. Cells were stored at -80°C until use. Cells were thawed and dissolved in lysis buffer (50 mM Sodium Phosphate (NaPO4) (Fisher), 0.3 M Sodium Chloride (NaCl) (Fisher), 10 mM imidazole (Fisher)), sonicated and centrifuged to collect inclusion bodies containing protein of interest. Inclusion bodies were dissolved and ground using a pestle and mortar into a chaotropic buffer (6M Guanidine Hydrochloride, (Melford), 0.1 M Sodium Phosphate (NaH2PO4) (Fisher), 10 mM tris (Fisher), pH 8) before sonication (50% intensity, 50% cycles, 8 minutes) to increase solubilisation. The expression plasmids are designed such that a hexahistidine tag is present at the N-terminal end of expressed DI, separated from it by a site for the protease Factor Xa (FXa)(*11*). The expressed protein from the solubilized inclusion bodies was therefore purified on a nickel column (which binds the hexahistidine tag), re-folded in 0.6M arginine buffer (pH 8.5) and dialysed against 20mM Tris, 0.1M NaCl, pH 8. Protein was again purified post-folding using a nickel column, dialysed against PBS and stored.

PEGylation:

Protein was reduced at a concentration of 0.4 mg/ml in 2M Arginine, 20 mM Sodium Phosphate (NaPO4, 0.1 M NaCl), 40 mM EDTA at pH 8.0 with 0.1M DTT for 1 hour at 20 °C. This was followed by removal of the reductant and buffer exchange on a PD -10 column to an identical buffer with 25 mM Arginine rather than 2M. PEGylation (TheraPEG, an Abzena Technology) reagent was added (1:0.8 molar ratio) and incubated for four hours at 4 °C. This was then buffer

exchanged to 20 mM Sodium acetate with 0.05% Tween at pH 6.0 for cation exchange purification on a 5 ml SP-HP column (GE Healthcare) with a linear gradient from 20% buffer containing 1M NaCl to 100 % of the same buffer at 2 ml/min for one hour. Fractions containing protein of the expected size of PEG-DI were identified by peaks on a chromatogram at 280nm and then pooled.

Cleavage with FXa to remove hexahistidine tag

Mono-PEGylated protein was buffer exchanged and cleaved as in McDonnell *et al*.(*15*). Briefly, proteins were dialysed against 20 mM HEPES buffer at a pH of 6.8, human FXa (Cambridge Bioscience) was added at a ratio of 1:100 with 1mM $CaCl_2$ and incubated at room temperature overnight. Purification of cleaved protein was carried out by cation exchange on an SP-HP column as above and buffer exchanged on a PD-10 column to PBS.

Chemical characterisation:

Proteins were characterised for purity by reverse phase high performance liquid chromatography (RP-HPLC) using a C8 column with a linear gradient between 2% Acetonitrile (AcN) , 0.05% trifluoroacetic acid (TFA) and 100% AcN 0.065% TFA. Proteins were also characterised by SDS PAGE for size.

Production of APS-IgG samples from serum of patients

In order to characterize the biological properties of the PEG-DI products we tested their ability to inhibit the effects of APS-IgG from 18 patients (10 with primary APS and 8 with SLE-associated APS) in three assays; binding to whole β2GPI in ELISA; a clotting assay that simulates the clinical lupus anticoagulant test; and stimulation of thrombosis in a mouse model. The clinical and serological data for the patients from whom these APS samples were obtained are shown in Table 1. IgG was purified from serum of these patients by passing these samples down a protein G

column (Peirce). Eluted IgG was neutralised with 500 µl of 1 M Tris solution. Samples were then dialysed against PBS and total IgG content was quantified using a BCA assay.

Competitive Inhibition anti-β2GPI ELISA

This was carried out as described previously(*15*). In brief, serum was diluted 1:50 in PBS/1% bovine serum albumin (BSA) and tested for binding in an anti-β2GPI ELISA. For each serum sample, the dilution level giving 50% of maximum binding in this assay was selected for use in the inhibition assay. Samples at this dilution were incubated with varying concentrations of a DI construct for two hours at room temperature then tested again in the anti-β2GPI ELISA. The results were plotted as "% Binding remaining" on the y-axis against concentration of inhibitor on the x-axis where "% Binding remaining" = (Binding in presence of inhibitor)/(Binding in absence of inhibitor) x 100.

Modified Direct Russell Viper Venom Test (dRVVT):

Although APS is characterized by increased clotting caused by aPL, one of the tests used in clinical practice to detect aPL in serum is called the lupus anticoagulant (LA) test. The rationale behind the test is that when clotting is triggered *in-vitro* using a reagent containing dilute Russell Viper Venom, the effect of adding aPL from a patient with APS is to inhibit clotting, prolonging the clotting time (this *in-vitro* effect is opposite to the *in-vivo* effect of the same antibodies, which is why this is called the lupus anticoagulant test). The prolongation cannot be reversed by adding serum from a healthy person (showing that it is not due to lack of a clotting factor) but is reversed by adding excess phospholipid. The result is therefore typically expressed as the ratio of dRVVT-stimulated clotting time of the patient's plasma in the absence of phospholipid (LA-sensitive reagent or LS) to the clotting time in the presence of phospholipid (LA-resistant reagent or LR). A ratio above 1.1 suggests the presence of LA. Several studies have suggested that the LA test is

particularly strongly linked to clinical outcomes, for example adverse pregnancy outcomes in patients with SLE.

We modified the dRVVT to test whether our PEG-DI products inhibit lupus-anticoagulant effects of APS-IgG. We used a standard lupus anticoagulant kit containing LS and LR dRVVT reagents. Clotting was measured using a CA-50 coagulometer (Sysmex). We purified APS-IgG from the serum of patients whose plasma was known to test positive for LA using the dRVVT test. APS-IgG samples were added to commercially available healthy human plasma at a concentration of 500mcg/ml for 15 minutes at 37 °C before testing in the LA assay. Those APS-IgG that gave LS/LR ratios > 1.1 were used for inhibition assays. APS-IgG was incubated with inhibitor (DI or PEG-DI) at a 1:1 molar ratio with 50 µl of plasma for 15 minutes at 37°C. This mixture was then added to 350 µl of plasma and re-incubated for 15 minutes before testing for LA effect. The outcome measure was ratio of clotting times seen in the presence of LS and LR reagents (LS/LR ratio). Reduction in this LS/LR ratio in the presence of DI or PEG-DI signified an inhibitory effect on the LA action of APS-IgG. In control experiments, we used octreotide (a kind gift from Dr Kozakowska of PolyTherics) or albumin (Sigma Aldrich) instead of DI or PEG-DI to exclude a non-specific effect of adding extraneous proteins to this assay system.

Passive transfer mouse model

The method was as described in previous papers(*16*). IgG from APS patient serum (APS-IgG) was purified and quantified as previously stated and IgG from normal human serum (NHS-IgG) was produced in a similar fashion. Male CD-1 mice (n=5 per group) (Charles River Laboratories, Wilmington, MA) between 6 to 8 weeks in age were injected intraperitoneally (IP) with 500mcg APS-IgG and then 30-60 minutes later with either DI, PEG-DI conjugate or PBS control. Negative control mice were injected IP with 500mcg NHS-IgG. All materials had endotoxin levels below 1.5 EU/ml. These injections were repeated at 48 hours after the first injection and the

thrombogenicity of aPL was assessed in a mouse model of induced thrombosis at 72 hours after the first injection. At this time, mice were anaesthetized and one of the femoral veins was exposed for observation and an approximate 0.5mm segment was trans-illuminated using a microscope equipped with a closed-circuit video system. The isolated vein segment was pinched to introduce a standardized injury and thrombus formation and dissolution was visualized and recorded. The treatment groups were as follows: APS-IgG + PBS control, NHS-IgG Alone, APS-IgG + 40 mcg non-PEGylated DI, APS-IgG + 40 mcg non- PEGylated D8S,D9G, APS-IgG + 40 mcg 20 kDa PEGylated DI, APS-IgG + 40 mcg 20 kDa PEGylated D8S,D9G, APS-IgG + 20 mcg 20 kDa PEGylated DI, PEG alone and APS-IgG + PEG.

Three outcome measures were assessed, as fully described in previous papers(*16*). These were
a) Induced thrombus size: A total of 3 thrombi were generated in each mouse and the largest cross-sectional area of each thrombus during the formation-dissolution cycle was measured 5 times and a mean value calculated (in μm^2).
b) Activity of tissue factor (TF) in peritoneal macrophages. After measurement of thrombus area, peritoneal macrophages were collected by flushing the abdominal cavity with 5ml PBS, which was retrieved after vigorous abdominal massage. The peritoneal fluid obtained was processed in a manner previously described to remove red blood cells and dead cells and to isolate macrophages following centrifugation, resuspension and sonication in a Tris-saline buffer and lysates were stored at -80°C until use. The TF activity was measured using a commercial chromogenic assay (American Diagnostica, Carlsbad, CA, USA) that measures FXa activity after activation by the TF-Factor VII complex. Results were standardized with reference to the protein concentration of lysates and expressed in pM/mg/ml.
c) Tissue Factor expression in mouse carotid: Subsequent to peritoneal fluid removal, pieces of approximately 5 mm of uninjured carotid arteries were dissected from both sides in each animal. These dissected carotids were immediately submerged in Tris buffered saline containing heparin

and aprotinin protease inhibitor (Sigma Aldrich) and homogenized in an ice bath. The homogenate was subsequently centrifuged, the pellet resuspended, sonicated and stored at -80°C until use. The TF activity of the homogenate was measured as described above.

All animals were housed in the viral antibody-free barrier facility at the University of Texas Medical Branch. Animal use and care were in accordance with the UTMB Institutional Animal Care and Use Committee (IACUC) guidelines.

Statistical analysis

Results were expressed as means plus or minus standard deviation as appropriate. A one way analysis of variance by ANOVA followed by the Tukey-multicomparison test was used to compare differences among mouse groups. These analyses were performed using the xlStat. Statistics were also carried out in prism using ANOVA and T Tests.
